# Development of an assay pipeline for the discovery of novel small molecule inhibitors of human glutathione peroxidases GPX1 and GPX4

**DOI:** 10.1016/j.redox.2023.102719

**Published:** 2023-05-16

**Authors:** Dorian M. Cheff, Qing Cheng, Hui Guo, Jameson Travers, Carleen Klumpp-Thomas, Min Shen, Elias S.J. Arnér, Matthew D. Hall

**Affiliations:** a Early Translation Branch, National Center for Advancing Translational Sciences, National Institute of Health, 9800 Medical Center Drive, Rockville, MD, 20850, United States; b Division of Biochemistry, Department of Medical Biochemistry and Biophysics, Karolinska Institutet, SE, 171 77, Stockholm, Sweden; c Department of Selenoprotein Research and the National Tumor Biology Laboratory, National Institute of Oncology, Budapest, Hungary

**Keywords:** Glutathione peroxidase, Selenoprotein, High-throughput screening, Reactive oxygen species, Drug discovery

## Abstract

Selenoprotein glutathione peroxidases (GPX), like ubiquitously expressed GPX1 and the ferroptosis modulator GPX4, enact antioxidant activities by reducing hydroperoxides using glutathione. Overexpression of these enzymes is common in cancer and can be associated with the development of resistance to chemotherapy. GPX1 and GPX4 inhibitors have thus shown promise as anti-cancer agents, and targeting other GPX isoforms may prove equally beneficial. Existing inhibitors are often promiscuous, or modulate GPXs only indirectly, so novel direct inhibitors identified through screening against GPX1 and GPX4 could be valuable. Here, we developed optimized glutathione reductase (GR)-coupled GPX assays for the biochemical high-throughput screen (HTS) of almost 12,000 compounds with proposed mechanisms of action. Initial hits were triaged using a GR counter-screen, assessed for isoform specificity against an additional GPX isoform, GPX2, and were assessed for general selenocysteine-targeting activity using a thioredoxin reductase (TXNRD1) assay. Importantly, 70% of the GPX1 inhibitors identified in the primary screen, including several cephalosporin antibiotics, were found to also inhibit TXNRD1, while auranofin, previously known as a TXNRD1 inhibitor, also inhibited GPX1 (but not GPX4). Additionally, every GPX1 inhibitor identified (including omapatrilat, tenatoprazole, cefoxitin and ceftibuten) showed similar inhibitory activity against GPX2. Some compounds inhibiting GPX4 but not GPX1 or GPX2, also inhibited TXNRD1 (26%). Compounds only inhibiting GPX4 included pranlukast sodium hydrate, lusutrombopag, brilanestrant, simeprevir, grazoprevir (MK-5172), paritaprevir, navitoclax, venetoclax and VU0661013. Two compounds (metamizole sodium and isoniazid sodium methanesulfate) inhibited all three GPXs but not TXNRD1, while 2,3-dimercaptopropanesulfonate, PI4KIII beta inhibitor 3, SCE-2174 and cefotetan sodium inhibited all tested selenoproteins (but not GR). The detected overlaps in chemical space suggest that the counter screens introduced here should be imperative for identification of specific GPX inhibitors. With this approach, we could indeed identify novel GPX1/GPX2- or GPX4-specific inhibitors, thus presenting a validated pipeline for future identification of specific selenoprotein-targeting agents. Our study also identified GPX1/GPX2, GPX4 and/or TXNRD1 as targets for several previously developed pharmacologically active compounds.

## 1 Introduction

**Fig. 1:**
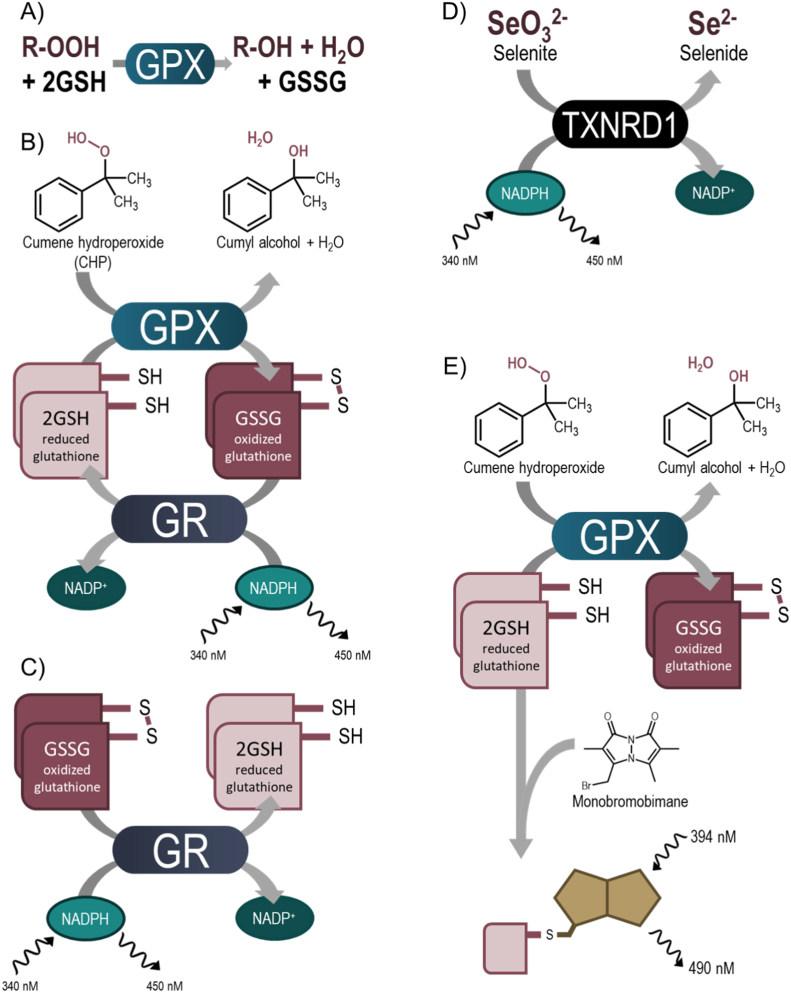
**Assay diagrams.** A) GPX activity schematic; B) Schematic diagram of fluorescence-based high-throughput screening assay used to evaluate GPX activity. GPX enzymatic reaction when reducing CHP is coupled to GR with the detection of NADPH fluorescence. C) Schematic diagram of GR inhibition assay used as a counter-screen to identify small molecules that show inhibition in the primary screening assay via indirect inhibition of the coupling enzyme; D) Schematic diagram of TXNRD1 activity assay reducing selenite to selenide in an NADPH-dependent reaction, used as counter-screen to identify small molecules with broader Sec-targeting activity; E) Schematic diagram of orthogonal endpoint GPX inhibition assay. Following a reaction window, monobromobimane is added and reacts with the thiols in remaining GSH to quantify GPX activity.

Many cancer cell types have higher basal levels of reactive oxygen species (ROS) production, and altered expression of antioxidant enzymes, when compared to normal cells. At moderate levels, ROS, like hydrogen peroxide and the hydroxyl radical, can cause substantial cellular damage by reacting with DNA, lipid membranes, and proteins, leading to tumorigenesis [1,2]. At high levels, however, different hydroperoxide species can contribute to several cell death pathways, and many anticancer therapeutics function by targeting and perpetuating imbalance in these redox systems [[3], [4], [5], [6]]. Selenoproteins, such as the glutathione peroxidase (GPX) family, protect cells from increased levels of hydroperoxides and oxidative stress, although individual GPXs can have diverse roles in different cellular contexts [7]. GPXs couple the reduction of hydrogen peroxide and other organic hydroperoxides with the oxidization of glutathione (GSH) to glutathione disulfide (GSSG) (Fig. 1A) [[8], [9], [10]]. Many cancer types show an upregulation of GPXs to compensate for increased hydroperoxide levels, or as a function of altered signaling, and these important enzymes have been described as modulators of metastases [7,[11], [12], [13]]. The three primary isoforms, GPX1, GPX2, and GPX4, differ in expression and substrate specificity. The most abundant isoform, GPX1, is a homotetramer and can reduce a range of hydroperoxides [8,14]. GPX2 has high homology with GPX1 and can reduce similar substrates, albeit with a significantly reduced turnover, but is expressed primarily in epithelial cells [8]. GPX4 is monomeric and unique in its ability to reduce lipid hydroperoxides [8,9]. GPX4 has recently been identified as an important regulator of a non-apoptotic form of iron-dependent cell death, ferroptosis, which is of interest as a novel anticancer pathway [15,16]. Inhibition of GPX activity, using small molecules or cellular knock-down models, can trigger repressed tumor growth and cell death [17,18]. Additionally, GPX1, GPX2, and GPX4 expression is upregulated in cancer cells resistant to many chemotherapeutics, and many treatment-resistant cells have been found to accumulate lipid peroxides, showing an increased dependence on the antioxidant activity of GPX4 [[19], [20], [21], [22], [23], [24]]. The relationship between GPX1 and resistance remains less clear and appears to have some tissue-dependence; with increased activity promoting resistance in Non-Small Cell Lung Cancer (NSCLC) and breast carcinoma while lower GPX1 expression drives gemcitabine resistance in pancreatic ductal adenocarcinoma (PDAC) [[25], [26], [27]]. GPX2 overexpression has been characterized in several cancers such as colorectal adenomas and carcinomas, as well as promoting progression of lung adenocarcinomas [[28], [29], [30]]. Though, the effects of the upregulation of GPXs are not entirely clear; in some cases (and with some genetic variants) this is associated with improved prognosis, tumor suppression, and even increased sensitivity to chemotherapeutics, while several cancer types instead exhibit decreases in GPX expression [[30], [31], [32], [33]].

Because of the role of glutathione peroxidases in controlling hydroperoxide levels, counteracting oxidative stress, and regulating cell death pathways, inhibitors of GPXs have promising potential as therapeutics for several malignancies. Specific inhibitors will be essential to fully characterize the effects of GPX inhibition, and to validate the relevance of isoform specific targeting as anti-cancer treatments. However, due to the technical difficulties in producing active selenocysteine (Sec)-containing recombinant GPXs due to highly intricate selenocysteine translation machineries, large screening efforts have not been attempted and very few specific drug-like inhibitors have been identified [[34], [35], [36]].

To date, very few GPX1-specific inhibitors have been described. A novel class of tri- and tetracyclic pentathiepins have been recently shown to inhibit GPX1 and not induce ferroptosis, though direct measurement of GPX4 modulation was not performed [36]. Pan-GPX inhibitors, however, fall into two categories: GSH competitors, and selenol-interacting agents. Agents that inhibit cellular GSH synthesis, such as erastin and l-buthionine sulfoxamine (BSO), or selenoprotein translation machinery inhibitors, such as statins, are indirect inhibitors of GPX activity [[37], [38], [39]]. Mercaptosuccinic acid (MSA) and some thiol-containing peptides can bind to and inhibit the activity of GPXs, and methylmercury, as well as some lead-containing compounds, can react irreversibly with the Sec residue of GPX [40]. Inhibition of selenoproteins by metals is thought to contribute to the metal-related toxicity of these agents. Additionally, the FDA approved arthritis treatment auranofin (AF, Ridaura®) is known to inhibit the selenoproteins thioredoxin reductase (TXNRD1) and GPX1 with emerging potential as an anti-cancer agent [6,41,42]. Biochemical inhibition by auranofin has not been observed with GPX4, a monomeric isoform, but recent studies have shown decreased GPX4 expression in mutant p53 non-small cell lung cancer following auranofin treatment [41,43].

The integral role of GPX4 in reducing lipid hydroperoxides, and as a regulator of ferroptosis, has instigated interest in the development of GPX4-specific inhibitors. A high-throughput oncogenic-RAS synthetic lethal screen identified (1*S*,3*R*)-RSL3 (referred to as RSL3 in this paper) as a mutant-RAS selective compound that induces the accumulation of lipid hydroperoxides characteristic of ferroptosis [44]. This form of cell death is preventable with iron chelators, such as DFO, or antioxidant ferroptosis inhibiting compounds, such as ferrostatin-1 [15].Two additional small molecules, ML162, and ML210, were identified in the same screen, and all three molecules were subsequently determined to induce ferroptosis with a mechanism consistent with binding of GPX4 in a lysate pull-down assay [16]. Additional studies have described in more detail the covalent binding of these ferroptosis inducing (FIN) compounds to GPX4 and leading to subsequent degradation of the enzyme in cells [35,45]. However, direct biochemical inhibition of Sec-containing GPX4 by RSL3 has not been demonstrated, and it was proposed that cellular inactivation requires an adaptor protein, 14-3-3ε [46]. ML210, similarly, has been shown to require a cellular context to enact GPX4 inhibition [47]. Furthermore, we have recently described the potent biochemical and cellular inhibition of TXNRD1, but not of pure GPX4, by RSL3 or ML162 [42]. Thus, the existing small molecule inhibitors of GPXs are not ideal for further development as GPX-targeting compounds and additional discovery programs are needed to properly probe either GPX1 or GPX4.

Using novel methods to express and purify recombinant Sec-containing GPXs we have produced large amounts of purified enzymatically active GPX1 and GPX4 [[48], [49], [50]], and have here developed and miniaturized assays for these enzymes amenable to high-throughput small molecule screening. We have used these assays to screen almost 12,000 small molecules in mechanistically annotated libraries to identify compounds with low micromolar inhibitory activity against GPX1 and/or GPX4. These libraries include approved drugs (USA, Europe, Japan, Australia, and Canada), investigational drugs, novel agents entering the clinic, and other pharmacologically active probe compounds. Using a new panel of activity screens, counter-screens, and orthogonal assays to determine mode of inhibition, we have thus identified GPX-targeting compounds, and optimized a pipeline to enable future discovery of additional potent and specific inhibitors of GPXs.

## 2 Methods

### 2.1 Chemicals, reagents, and libraries

Tris(hydroxymethyl)aminomethane (Tris-HCl; T3253), ethylenediaminetetraacetic acid (EDTA; 324504), sodium chloride solution (NaCl; S5150), l-glutathione reduced (GSH, G4251), l-glutathione oxidized (GSSG; G4501), cumene hydroperoxide (CHP; 247502), β-nicotinamide adenine dinucleotide 2′-phosphate reduced tetrasodium salt hydrate (NADPH; N7505), monobromobimane (mBBr; B4380), and bovine serum albumin (BSA; A3311) were purchased from Sigma Aldrich (St. Louis, MO). Dimethyl sulfoxide (DMSO; BP231) was purchased from ThermoFisher (Pittsburgh, PA).

The primary screening was completed using the Library of 1280 Pharmacologically Active compounds (LOPAC®^1280^) from Sigma-Aldrich (St. Louis, MO), and three internal mechanistically annotated libraries; the Mechanism Interrogation Plate (MIPE) 5.0, the NCATS Pharmaceutical Collection (NPC) 2.0, and the NCATS Pharmacologically Active Chemical Toolbox (NPACT), for a total of 11,892 compounds. The compound plates were prepared as described previously [51]. Purified recombinant human GPX1, GPX2, GPX4, GR, and TXNRD1 were produced and purified as previously reported [[48], [49], [50]]. As production of GPX2 was not optimized at the onset of screening, only GPX1 and GPX4 underwent library screening, and GPX2 was used as a follow-up.

### 2.2 Quantitative high-throughput biochemical screening

Compounds from the MIPE 5.0, NPC 2.0, and NPACT libraries were screened at seven concentrations (LOPAC was run at five concentrations) ranging from 68.3 nM to 49.8 μM. All HTS confirmatory, orthogonal, and counter-screen assays were tested in 11-point dose-response ranging from 842.5 pM to 49.8 μM. Follow-up and orthogonal assays were run in quadruplicate unless otherwise stated. Assay schemes are illustrated in Fig. 1.

#### 2.2.1 Primary GR-coupled GPX activity assays

To measure GPX activity, assays were performed in 1536-well format in black medium-binding solid-bottom plates (Greiner). These assay conditions were optimized for high-throughput screening from an assay first described by Paglia and Valentine [52]. GPX activity is measured indirectly by coupling the reaction with GR whereby GPX reduces hydroperoxide by oxidizing two GSH to GSSG and the oxidized glutathione is recycled to its reduced state by GR and NADPH (Fig. 1B). The rate of consumption of NADPH is directly proportional to the activity of GPX and can be measured by the decrease in fluorescence at 340 nm/450 nm.

First, 3 μL of enzyme in TES assay buffer (50 mM Tris-HCl, 2 mM EDTA (pH 7.5), 150 mM NaCl with enzyme [10 nM GPX1, 200 nM GPX4 ] and BSA [0.01%]) was added to columns 3–48 of 1536-well plates using a BioRAPTR Flying Reagent Dispenser (Let's Go Robotics (LGR), Carlsbad, CA). A no-enzyme control (0.01% BSA in TES assay buffer) was added to columns 1–2. Compounds (25 nL, library and DMSO controls) were added to each well with a pin tool (Kalypsys), and the plates were incubated for 30 min at room temperature before 1 μL of master mix (100 nM GR, 1 mM GSH, 0.5 mM NADPH in TES buffer) was added. 1 μL of cumene hydroperoxide (CHP) ([0.5 mM] in 50% EtOH) was then added within 5 min to initiate the reaction. Fluorescence at 340/450 nm was measured using a ViewLux multimodal detector (PerkinElmer, Waltham, MA) at initial t = 0 and endpoint t = 15 min (GPX1) or t = 20 min (GPX4).

Follow-up confirmation assays were also run in 96-well format using medium binding clear 96-well SpectraPlate microplates (PerkinElmer) and NADPH absorbance at 340 nm was measured every 30 s for 30 min using an Infinite® M Nano (Tecan).

#### 2.2.2 GR counter-assay

To identify false positive compounds that interfere with the primary assay through inhibition of GR, ‘hits’ from the primary screening were run against a GR counter assay to identify this possible off-target inhibition (Fig. 1C). Briefly, 3 μL enzyme mixture (100 nM GR, 0.01% BSA in TES buffer) was dispensed into columns 2–48 of a 1536-well black medium-binding solid-bottom plates. The no-enzyme control (0.01% BSA in TES buffer) was included in column 1 and 2. Compounds and controls (23 nL) were added to each well with a pin tool (Kalypsys), and the plates were incubated for 30 min before 2 μL of substrate mixture (1 mM GSSG 0.4 mM NADPH in TES buffer) was added to each well and absorbance at 340 nm was measured at t = 0 and t = 20 min using a ViewLux Multimodal detector (PerkinElmer).

#### 2.2.3 Monobromobimane orthogonal assay

As an orthogonal assessment of GPX inhibition, a monobromobimane endpoint assay was used (Fig. 1E). Briefly, 30 μL GPX mixture (0.01% BSA and enzyme [1 nM GPX1 or 25 nM GPX4] in TES buffer) was added to columns 2–24 of 384-well black medium-binding plates (Greiner) using a BioRAPTR FRD. TES buffer was used as a no-enzyme control in column 1. Compounds and controls were added in 11-point dose-response titration and incubated with enzyme for 30 min whereupon 10 μL of 400 μM GSH in TES buffer was added before starting the reaction with the addition of 10 μL 200 μM CHP and the reaction was left at room temperature for 10 min (GPX1) or 15 min (GPX4). Upon GPX activity, GSH was depleted over time, whereupon 500 μM mBBr was added and fluorescence at 394/490 was measured using a Viewlux multimodal detector every 20 s for 20 min.

#### 2.2.4 Thioredoxin reductase 1 (TXNRD1) inhibition assay

TXNRD1 activity was assessed using a modified 1536-well TXNRD1 inhibition assay, based upon TXNRD1-catalyzed selenite reduction as previously described (Fig. 1D) [53]. Briefly, 2 μL of human TXNRD1 mixture in TE buffer (50 mM Tris-HCl, pH 7.5, 2 mM EDTA, 180 nM TXNRD1 and 0.01% BSA) followed by 1 μL of NADPH in TE buffer were dispensed into each well of a Greiner black solid-bottom 1536-well plates using a BioRAPTR 2.0 (Let's Go Robotics (LGR)) flying reagent dispenser. TE buffer was dispensed into column 1 and 2 as a no-enzyme control. Finally, 1 μL of sodium selenite in TE buffer was dispensed into each well for final concentrations of 90 nM hTXNRD1, 0.4 mM NADPH, and 0.4 mM sodium selenite. The plate was read in a Viewlux multimodal detector in kinetic mode and fluorescence at 340 nm/450 nm was measured every minute for 8 min.

#### 2.2.5 Nano differential scanning fluorimetry (nanoDSF) characterization

Thermal stabilization of enzyme ± DMSO or ± compound was carried out in a Prometheus NT.48 instrument (NanoTemper Technologies) with an excitation wavelength of 280 nm. Intrinsic fluorescence at 330 nm and 350 nm was monitored while heating. Capillaries were filled with ∼10 μL of a solution of GPX1/4 (1 mg/mL), ± small molecule (100 μM) or DMSO, in TES buffer and placed into the instrument. The capillaries were subjected to a temperature range from 30 to 90 °C at a ramp rate of 2 °C/min. The ratio of the recorded emission intensities (350nm/330 nm) were plotted as a function of temperature. This ratio and first derivatives were calculated using the NanoTemper software and were plotted using GraphPad Prism.

### 2.3 Analysis and triage

Percent activity was determined by first calculating delta RFU (decrease in fluorescence between t = 0 and t = 20/30 min in primary GPX, GR counter-screen, and TXNRD1 assays, or increase in fluorescence between t = 0 and t = 20 min in the Monobromobimane GPX endpoint assay), and normalizing enzyme activity using median delta RFU values for DMSO-treated enzyme control, and DMSO-treated no-enzyme controls.

Compound inhibitory activity in the qHTS assays was determined by plotting dose-response data for each sample and fitting with a four-parameter logistic fit to assess IC_50_ values, efficacy (maximal response), and AUC (area under the curve), as previously described [51]. Hits were classified by the quality of the concentration-response curves (CRC). Curve classes −1.1 and −1.2 being the highest-confidence actives with complete CRCs and classes −2.1 and −2.2 being lower-confidence actives with incomplete CRCs. Compounds in these classes were further categorized as high- and low-quality actives based on maximal responses of ≤25% or ≤50% remaining activity, respectively, and an IC_50_ value of ≤20 μM.

Compounds were further clustered and analyzed using TIBCO Spotfire 11.4.4 (Spotfire Inc., Cambridge, MA, USA) based on activity from both primary and follow-up screens, and figures and final curves were generated using log(inhibitor) vs. response for four-parameter variable slope GraphPad Prism version 9.3.1 (GraphPad Software, San Diego, CA, USA).

HTS data and assay information can be found at PubChem using ID numbers 1845191 for the GPX1 HTS and 1845192 for the GPX4 HTS.

## 3 Results

### 3.1 Assay development and optimization

**Fig. 2:**
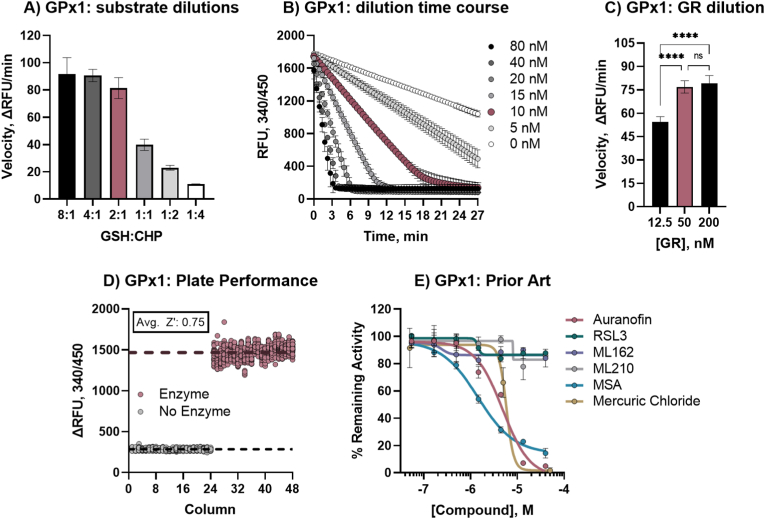
**Validation of GPX1 qHTS primary assay.** A) GPX1 assay velocity with 10 nM GPX1 and 0.5 mM CHP with varying GSH concentrations, red denotes optimized 2:1 ratio of 1 mM GSH and 0.5 mM CHP (8:1, 4 mM GSH; 4:1, 2 mM GSH; 2:1, 1 mM GSH; 1:1, 0.5 mM GSH; 1:2, 0.25 mM GSH; 1:4, 0.125 mM GSH); B) GPX1 dilution and time course of GR-coupled activity assay with 100 nM GR, 1 mM GSH, 0.5 mM CHP, and 0.5 mM NADPH, red denotes optimized concentration of 10 nM; C) effect of GR concentration on GPX1 velocity with optimized substrate and enzyme conditions, red bar denotes optimized concentration of 50 nM; D) 1536-well plate performance of optimized GR-coupled GPX1 assay, average Z′ for whole plate was 0.75; E) Prior art GPX1 inhibitor activities in optimized assay curve fit for IC50 calculation: IC_50_ of auranofin, MSA, and mercuric(II) chloride were 4.84 μM, 1.44 μM, and 5.83 μM, respectively. (For interpretation of the references to color in this figure legend, the reader is referred to the Web version of this article.)

**Fig. 3:**
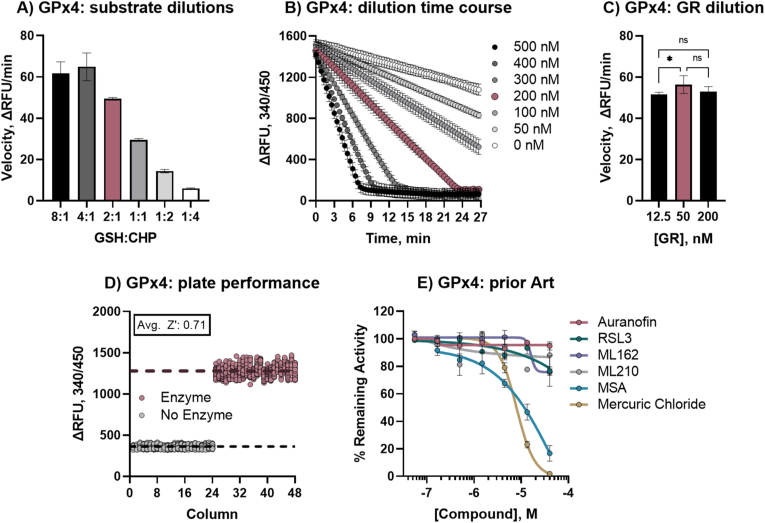
**Validation of GPX4 qHTS primary assay.** A) GPX4 assay velocity with 200 nM GPX4 and 0.5 mM CHP with varying GSH concentrations, red denotes optimized 2:1 ratio of 1 mM GSH and 0.5 mM CHP (8:1, 4 mM GSH; 4:1, 2 mM GSH; 2:1, 1 mM GSH; 1:1, 0.5 mM GSH; 1:2, 0.25 mM GSH; 1:4, 0.125 mM GSH); B) GPX4 dilution and time course of GR-coupled activity assay with 100 nM GR, 1 mM GSH, 0.5 mM CHP, and 0.5 mM NADPH, red denotes optimized concentration of 200 nM; C) effect of GR concentration on GPX4 velocity with optimized substrate and enzyme conditions, red bar denotes optimized concentration of 50 nM; D) 1536-well plate performance of optimized GR-coupled GPX4 assay, average Z′ for whole plate was 0.75; E) Prior art GPX4 inhibitor activities in optimized assay curve fit for IC50 calculation: IC_50_ of MSA, and mercuric(II) chloride were 57.74 μM, and 7.90 μM, respectively. None of the Class II FINs showed inhibitory activity, as reported elsewhere [42]. (For interpretation of the references to color in this figure legend, the reader is referred to the Web version of this article.)

Modifying a previously described assay [52] to be amenable for high-throughput screening, GSSG production by GPXs was coupled to GR activity (Fig. 1B). This assay setup includes two human enzymes, GPX (either GPX1 or GPX4) and GR, that naturally function together in cellular responses to oxidative stress. GPX converts two GSH molecules to GSSG as it reduces a hydroperoxide, and as GR recycles GSSG to GSH, it consumes NADPH in the process. The NADPH can be directly measured by NADPH autofluorescence, providing a coupled readout of GPX activity wherein reduction of CHP is equivalent to the consumption of NADPH. Although both GPX1 and GPX4 efficiently reduce a variety of substrates, CHP was selected for a lower background activity when compared to H_2_O_2_ (Supp. Fig. 1). Initial titration of NADPH and GR in our miniaturized assay setup determined that maximal turnover and maximal signal window occurred at 500 μM NADPH and 100 nM GR (not shown) and 0.5 mM CHP was selected as substrate. Because GPXs do not follow Michaelis-Menten kinetics, an initial ‘matrix’ of GSH:CHP ratios was performed to determine good assay behavior. With the optimized GR and NADPH conditions, and 0.5 mM CHP, we tested several GSH concentrations from 0.125 mM to 8 mM, and in both GPX1 and GPX4 assays the 2:1 ratio resulted in optimal assay velocity (Fig. 2, Fig. 3A, optimized concentrations shown in red). GPX enzyme concentration was then optimized for reaction time, with 10 nM GPX1 and 200 nM GPX4 found to give suitable linearity in NADPH consumption during 15 min of incubation and complete NADPH consumption within 25 min (Fig. 2, Fig. 3B, optimized concentrations shown in red). As the concentration of GR in the GPX1 assay posed a potential for amassing GR-specific hits, additional GR concentration tests were conducted, ensuring that activity was dependent primarily on GPX activity, and not GR (Fig. 2, Fig. 3C). Initial assay performance was strong with average plate statistics of 0.75 Z′ and 6.8 S/B for GPX1 (Figs. 2D) and 0.71 Z′ and 7.8 S/B for GPX4 (Fig. 3D). Optimized assay conditions for GPX1 and GPX4 are displayed in Table 1.

**Table 1:** Protocol for GPX qHTS assays.

Step	Parameter	Value	Description
1	Dispense enzyme	3 μL	GPX1 or GPX4
2	Pin-transfer library compounds Pin-transfer controls	23 nL	1:3 dilution series 45–0.1 μL auranofin dilution series (GPX1)
3	Incubation time	30 min.	Room temperature
4	Dispense substrate A	1 μL	GSH/GR/NADPH
5	Dispense substrate B	1 μL	CHP
6	Read fluorescence	Endpoint	Viewlux, 340/450 nm

Step notes.

1. [1.67X] enzyme mix: 0.0133 μM hGPX1 (0.333 μL hGPX4), 0.0167% BSA in TES buffer (50 mM Tris-HCl, pH 7.5 + 2 mM EDTA + 150 mM NaCl); same buffer but no enzyme in column 1.

2. Pintool transfer (tip wash sequence: DMSO, IPA, 3 s vacuum dry); control compound dilution in column 2.

3. Lidded plate.

4. [5X] substrate A mix: 5 mM GSH, 0.05 μL hGR, 2.5 mM NADPH in TES buffer.

5. [5X] substrate B mix: 2.5 mM CHP in 50% EtOH.

6. 340 nm excitation, 450 nm emission, 2 s exposure, 10 flashes; t = 0 min and t = 15 min (20 min for GPX4).

Final conc.: 10 nM hGPX1 or 200 nM hGPX4, 0.01% BSA, 50 nM GR, 1 mM GSH, 0.5 mM NADPH, 0.5 mM CHP.

Previously reported inhibitors (‘prior art’) were selected and tested against the optimized assay. Of the five GPX inhibitors noted in the literature [16,42,54] only two compounds exhibited inhibitory activity on GPX1, Auranofin (IC_50_: 4.84 μM) and MSA (IC_50_: 1.44 μM) (Fig. 2E). MSA inhibited GPX4 with an IC_50_ of 57.7 μM. No previously reported GPX4-targeting compounds (RSL3, ML162, ML210) showed inhibitory activity in the GPX4 biochemical assay (Fig. 3E), as reported elsewhere in further detail [42]. To further ensure that the GPX4 assay was indeed inhibitable, mercuric chloride was assessed as a Sec-targeting agent [55], and showed inhibition with IC_50_ values of 5.83 μM and 7.89 μM for GPX1 and GPX4, respectively (Fig. 2, Fig. 3E).

The GR counter screen assay (Fig. 1C) was optimized for 1536-well format from a previous report [56]. GR concentration was optimized for an amenable reaction time (Supp. Fig. 2A) and GSSG concentration was optimized within the linear range of NADPH fluorescence (Supp. Fig. 2B). The ideal conditions were found using 25 nM hGR, 0.5 mM NADPH, and 200 μM GSSG. The linearity of the reaction suggested that imaging after a 15-min incubation would be sufficient. Assay performance was strong with average plate statistics of 0.77 Z’ and 34.9 S:B (Supp. Fig. 2C).

Monobromobimane (mBBr) is a heterocyclic compound that reacts readily with low molecular weight thiols, like glutathione [57]. In its reduced state, mBBr reacts with the thiol on glutathione, and becomes fluorescent. We here assessed whether mBBr would function as a suitable orthogonal endpoint measurement of GPX activity (Fig. 1D). We first assessed linearity and reaction speed and the signal window and rapid formation of the GSH-mBBr complex were found to be reproducible and suggested compatibility (Supp. Fig. 3A). However, due to the viscosity of the MBBr solution, liquid handling was better suited for a 384-well plate using larger dispense volumes. Using the mBBr:GSH ratio with the largest signal window and a 2:1 GSH:CHP ratio, GPX1 and GPX4 at several concentrations were tested at varying timepoints to assess the timing of non-coupled activity. The optimized assay conditions for GPX1 were found to be 5 nM hGPX1, 500 μM GSH, and 500 μM mBBr, while GPX4 required 50 nM hGPX4, 500 mM GSH, and 500 μM mBBr. Initial tests using the validated GPX1 inhibitor, auranofin, resulted in similar activity in either assay (IC_50_ of 1.94 μM in the GR-coupled assay, and 5.3 μM in the mBBr assay). The lower GSH concentrations required similar incubations, whereby mBBr was added to react with remaining GSH at t = 15 min for both enzymes (Supp. Fig. 3B). Performance for this assay was moderate with average plate statistics of 0.49 Z′ and a 1.5X S:B for GPX1 and 0.47 Z’ and a 1.8X S:B for GPX4 (Supp. Fig. 3C, Supp. Fig. 3D).

### 3.2 High-throughput screen

**Fig. 4:**
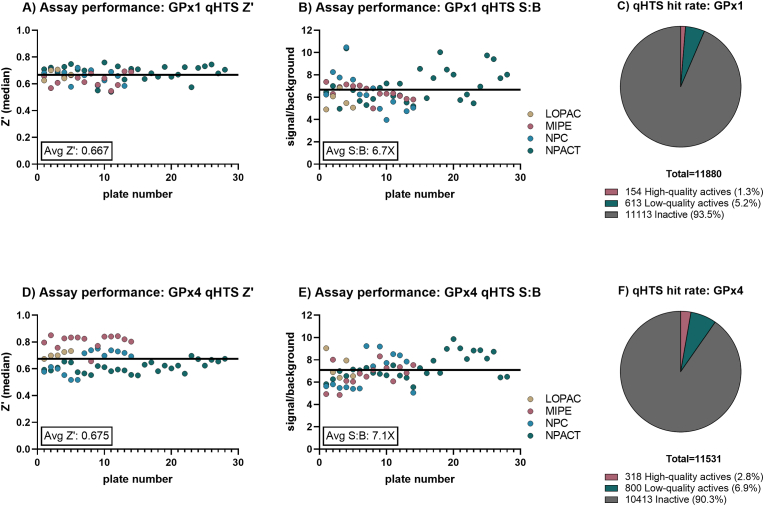
**Primary screening results and assay performance.** A) GPX1 qHTS plate performance as Z′ values for each library screened; B) GPX1 qHTS plate performance as signal:background calculations for each library screened; C) GPX1 qHTS hit rate; D) GPX4 qHTS plate performance as Z′ values for each library screened; E) GPX1 qHTS plate performance as signal:background calculations for each library screened; F) GPX4 qHTS hit rate.

Primary library screening using our principal assay (Fig. 1B) was run once using the LOPAC®^1280^ library at five concentration points, and the MIPE 5.0, NPC 2.0, and NPACT libraries at seven concentration points, totaling 11,892 unique small molecules, using an automated screening platform. During the screens, plates were read at t = 0 min and t = 15 or 20 min for GPX1 and GPX4, respectively. A total of 64 positive control (enzyme + DMSO treatment), and 64 negative control (no enzyme + DMSO treatment) wells were included in each 1536-well plate to monitor assay performance. The activity of each compound was measured as the change in signal from an initial (t = 0) read and was normalized against control wells. Using Δ15 min signal for GPX1, the HTS assays resulted in an average Z′ of 0.67 ± 0.05, and a S/B of 6.7 ± 1.4 (Fig. 4A–B). The GPX4 assay was run at Δ20 min and resulted in assay performance with an average Z’ of 0.67 ± 0.1 and a S/B of 7.1 ± 1.2 (Fig. 4D–E). High quality actives were defined as displaying >50% reduction in activity, an IC_50_ ≤ 20 μM, and a complete CRC (concentration–response curve). Analysis of the CRCs resulted in 180 high-quality GPX1 actives (Figs. 4C), and 318 high-quality GPX4 actives (Fig. 4F), with resulting hit rates of 1.3% and 2.7%, respectively.

Auranofin was present in the screening libraries and inhibited GPX1 with an IC_50_ of 2.26 μM and aurothioglucose (a known TRXRD1 inhibitor with GPX1 inhibitory activity) showed moderate inhibition of GPX1, reducing activity to 49% at the top dose (not shown).

### 3.3 Hit confirmation and triage

**Fig. 5:**
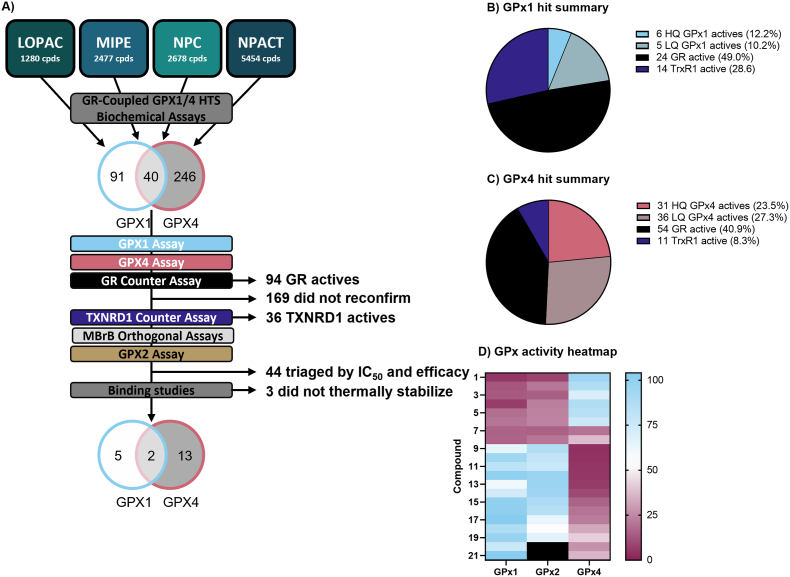
**Hit triage.** A) GPX1 and GPX4 HTS triage schematic; B) Summary of novel small molecule GPX1 actives in primary assay and counter-screens; C) Summary of novel small molecule GPX4 actives in primary assay and counter-screens; D) Heat map showing relationships of hit compounds based on the similarities of their activity profiles against GPX1, GPX2, and GPX4. For further details on each compound, see Table 2. Compounds 20 and 21 were not tested with GPX2 (shown as black box). Data is given as mean remaining activity at top concentration of *n* = 3 replicates.

The top compounds showing activity in the primary screen were reacquired, replated, and screened in an 11-point dose-response with freshly QC'ed stock to confirm activity. Of the 377 initial hits, 40 inhibited both GPX1 and GPX4 (‘pan-active,’ or ‘pan-GPX active’). Several secondary assays and counterscreens were selected to triage the initial screening hits, as summarized in the triage schematic in Fig. 5A. A total of 215 compounds from the primary assays reconfirmed activity in the expanded 11-point dose response validation, though 44 of these were removed for demonstrating activity at only the top concentration tested (i.e., weak activity). The 171 compounds remaining as confirmed underwent further analysis in secondary assays.

A GR activity assay was then run to identify ‘false positive’ compounds from the screen that interfered with the coupled primary assays. 94 compounds were found to inhibit the GR assay and were excluded from subsequent analysis. Of the 49 reconfirmed GPX1 hits, 24 (49%) of the compounds were GR actives, 54 of the 132 GPX4 actives showed activity in the GR assay (41%), and 59% of the pan-active hits were active in the GR assay (Fig. 5B–C).

The 121 compounds now remaining were subjected to further testing in a TXNRD1 activity assay to identify specific inhibitors of each GPX, pan-GPX inhibitors, and potentially also broad Sec-targeting compounds. Of the 121 GPX inhibiting compounds, 30 were found to inhibit TXNRD1 as well. This amounted to 14 of the 20 remaining GPX1 inhibitors and 11 of the remaining 42 GPX4 inhibitors (70% and 26%, respectively), and 5 of the compounds inhibiting both GPX1 and GPX4. We found four compounds that showed pan-GPX inhibition and TXNRD1 inhibition, but not GR, suggesting they may be specific Sec-targeting agents.

The 40 compounds remaining were then assessed for GPX activity in the orthogonal endpoint MBBr assay, and the inhibitory activity of all compounds tested was confirmed. Taken together, the assay panel suggested that the activities of these 40 compounds were indeed directly dependent on the GPXs, and not the coupling enzymes or assay formats (Fig. 5B–C).

A total of 5 GPX1-specific compounds, top 9 GPX4-specific compounds, and 2 pan-GPX active compounds (20 total, 21 with auranofin) were then assessed in the final assays.

### 3.4 Other glutathione peroxidases

Previous work has shown similarities in the substrate specificities of GPX1 and GPX2, and a more unique substrate profile of GPX4 [8]. The final compound set was thus assessed for inhibitory activity in a GPX2 assay to further delineate the specificity of the hits. The novel GPX4 inhibitors did not show significant inhibition of GPX2 (80.4 ± 14.2% remaining activity at top dose), while all GPX1 hits also showed potent inhibition of GPX2 (17.9 ± 6.4% activity remaining at top dose) (Fig. 5D).

**Fig. 6:**
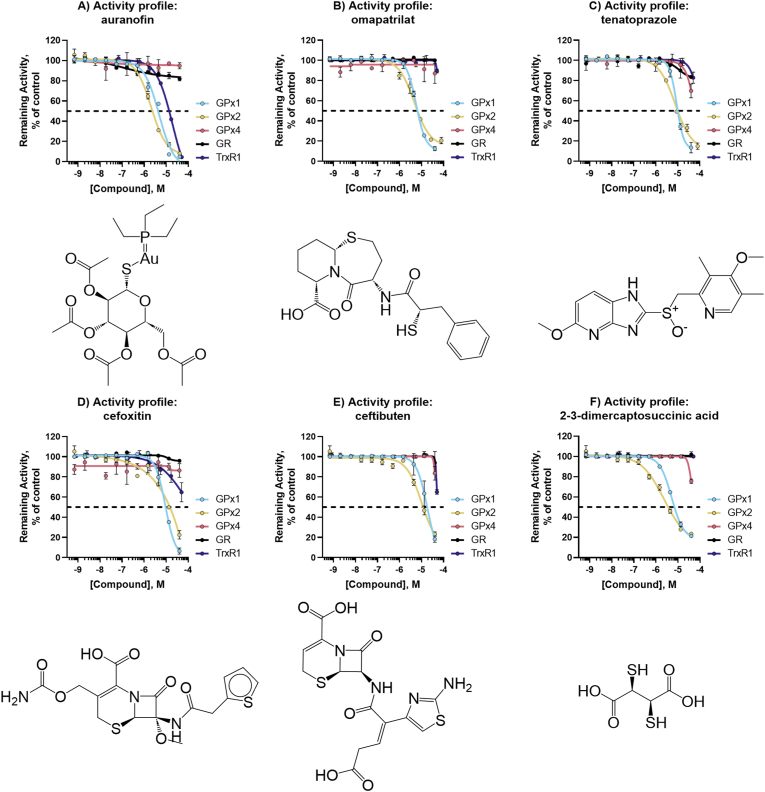
**Activity profiles of top GPX1 inhibitors.** Structure and activity profile of A) auranofin; B) omapatrilat; C) tenatoprazole; D) cefoxitin; E) ceftibuten; F) 2-3-dimercaptosuccinic acid. Data are normalized to DMSO and presented as mean ± s.d. of *n* = 3 replicates.

**Fig. 7:**
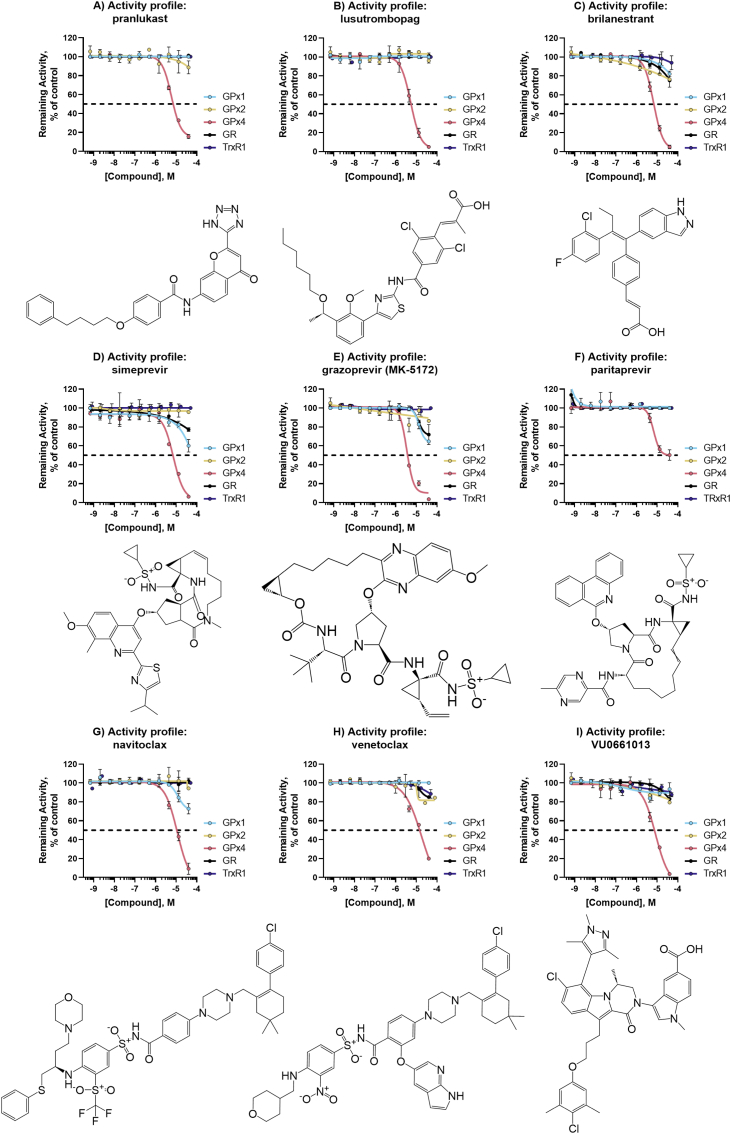
**Activity profiles of top GPX4 inhibitors.** Structure and activity profile of A) pranlukast sodium hydrate; B) lusutrombopag C) brilanestrant; D) simeprevir E) grazoprevir (MK-5172); F) paritaprevir; G) navitoclax; H) venetoclax; I) VU0661013. Data are normalized to DMSO and presented as mean ± s.d. of n = 3 replicates.

**Fig. 8:**
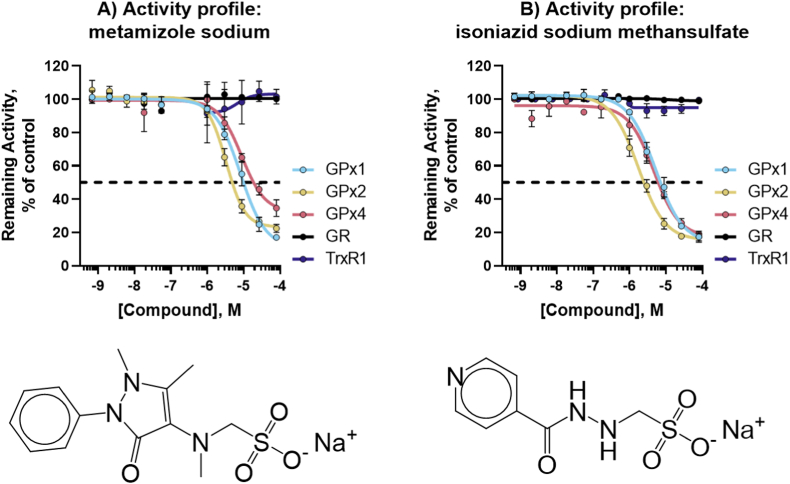
**Novel Pan-GPX inhibitors.** Structure and activity profile of A) metamizole sodium; B) isoniazid sodium methanesulfate; Data are normalized to DMSO and presented as mean ± s.d. of *n* = 3 replicates.

**Fig. 9:**
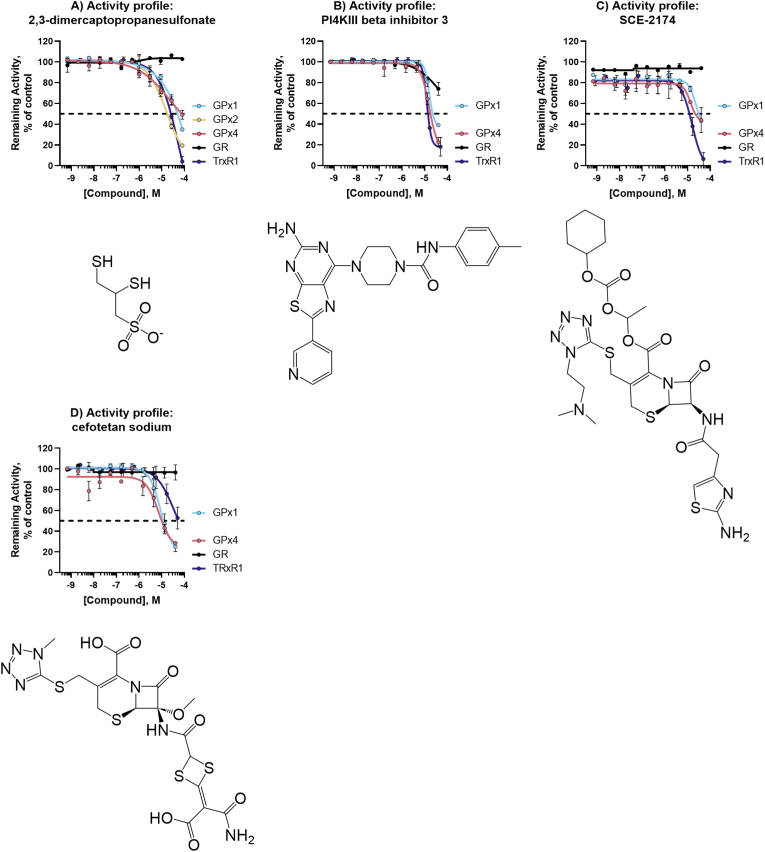
**Pan-selenoprotein inhibitors.** Structure and activity profile of A) 2,3-dimercaptopropanesulfonate; B) PI4KIII beta inhibitor 3; C) SCE-2174; D) cefotetan sodium; Data are normalized to DMSO and presented as mean ± s.d. of n = 3 replicates.

For complete activity profiles of the top novel inhibitors encompassing five GPX1 inhibitors (+ a reconfirmation of auranofin), nine GPX4 inhibitors, two pan-active GPX inhibitors, and pan-selenoprotein inhibitors see Fig. 6, Fig. 7, Fig. 8, Fig. 9.

### 3.5 Thermal stability using nanoscale differential scanning fluorimetry (nanoDSF)

**Fig. 10:**
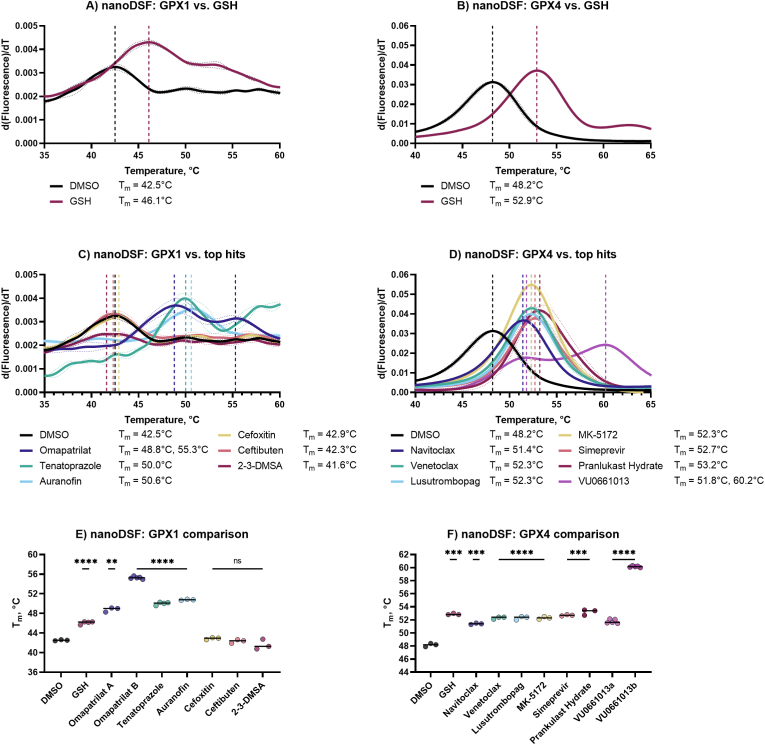
**Thermal stabilization of targets by hit compounds.** A) nanoDSF first derivative aggregation curves for GPX1 controls; B) nanoDSF first derivative aggregation curves for GPX4 controls; C) nanoDSF first derivative aggregation curves for GPX1 DMSO control and screening hits; D) nanoDSF first derivative aggregation curves for GPX4 DMSO control and screening hits; E) nanoDSF T_m_ summary for GPX1 controls and hits; F) nanoDSF T_m_ summary for GPX4 controls and hits. Data is presented as mean ± s.d. of *n* = 3 replicates, unless otherwise noted. Unpaired, two-tailed *t*-test; *P < 0.05, **P < 0.01, ***P < 0.001, ****P < 0.0001 in comparison to DMSO control.

We next aimed to evaluate via a biophysical method whether the compounds that inhibited GPX1, GPX4, or both GPX1 and GPX4 were interacting directly with the enzymes to further probe the interactions of the top inhibiting compounds with their target enzymes. nanoDSF is a high-throughput, label-free method to monitor changes in the fluorescence of intrinsic tryptophan in a protein as a function of temperature. Bound compounds typically thermally stabilize enzymes, resulting in a shift in melting temperature (T_m_) at which 50% of the enzyme is degraded [58,59]. GPX1 contains two tryptophans, which resulted in lower fluorescent signal than GPX4, which contains four. Glutathione was used as a substrate and known binder, resulting in a +3.6 °C increase in T_m_ for GPX1 (Fig. 10A) and +4.7 °C for GPX4 (Fig. 10B). The top performing inhibitors were next assessed for this cell-free binding to GPX1 and GPX4. With GPX1, auranofin showed a +8.3 °C shift in T_m_ to 50.6 °C from a DMSO control, tenatoprazole showed significant thermal stabilization with an increase of T_m_ of +7.5 °C, while omapatrilat treatment resulted in two inflection points, the first at +6.3 °C, and the second at +12.8 °C (Fig. 10C). No other top GPX1 hits showed thermal stabilization. Top GPX4 hits from our HTS showed thermal stabilization with T_m_ values ranging from +3.2 °C to +5.0 °C. VU0661013 showed two inflection points, at 51.8 °C and 60.2 °C, with T_m_ shifts of +3.6 °C and +12.0 °C, respectively (Fig. 10D). These effects were found to be statistically significant using a two-tailed Welch's *t*-test and were plotted by T_m_ (Fig. 10E and F).

**Table 2:**
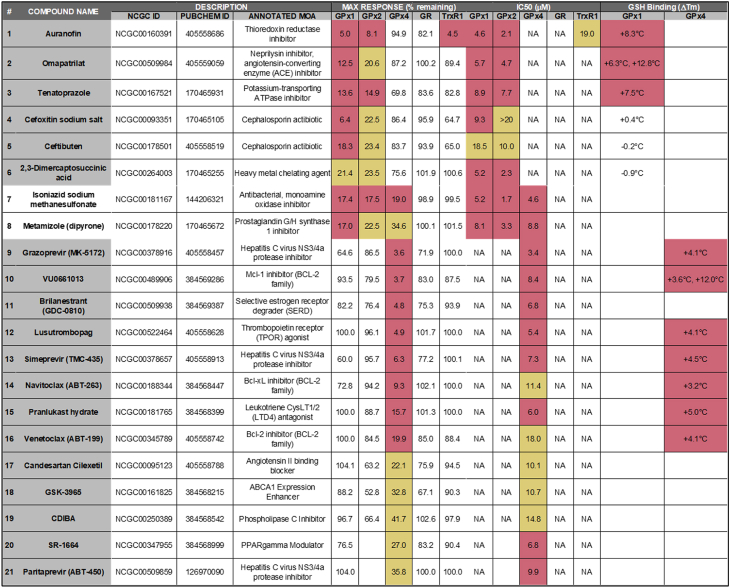
**Summary table of GPX inhibitors.** Compound Nr. corresponds to the heatmap in Fig. 5D. Max response at top concentration is reported as % remaining activity at top compound concentration tested, and IC_50_ of resulting CRC is reported for each enzymatic assay. Finally, shifts in nanoDSF melting temperature are given for the compounds tested. Red coloring denotes high-quality active characteristics, yellow denotes low-quality active characteristics, and no shading denotes inactivity. NA, not applicable.

## 4 Discussion

Here, we report the development and implementation of several primary and secondary assays in 1536-well format, as well as several characterization assays, for the identification of novel small molecule inhibitors of GPXs from high-throughput chemical libraries. These assays used Sec-containing enzymatically active human recombinant GPX1 and GPX4, produced and purified using a novel method in *E. coli*, to screen the mechanistically annotated compounds from our small molecule libraries (LOPAC^1280^, MIPE, NPC, and NPACT) in GR-coupled assays. All hits were additionally tested for inhibitory activity in a GR activity assay to remove any false positives from the primary screen, and subsequently also in TXNRD1 and GPX2 assays.

After screening 11,892 compounds against the optimized qHTS GPX1 and GPX4 assays, hit compounds were defined as having >50% inhibitory activity at the top concentration. Auranofin was present in three of the libraries screened and was a high-quality active hit for GPX1 in all three, representing the high reproducibility of this assay. RSL3 and ML210 were present in the HTS libraries, but as expected were not active hits in either screen [42]. Of the initial qHTS hits, 70% either did not reconfirm activity in the 11-point dose response follow-up GPX assays, or were shown to be false positives by inhibiting the coupled enzyme, GR.

Of the novel GPX4 inhibitors we found in the HTS, only 8% showed cross-inhibition with TXNRD1, suggesting the promiscuity of the FINs (RSL3 and ML210) may not be representative of future development of specific GPX4 inhibitors. Surprisingly, almost a third of all GPX1-specific inhibitors also showed high-quality inhibition of TXNRD1, which suggests a much larger overlap in chemical space for inhibition of these two enzymes, or alternatively a common mechanism of action against the Sec residue. If the latter is the case, this suggests that the Sec residue of GPX1 is more accessible to compounds than that in GPX4. It is well known that the Sec residue of TXNRD1 is solvent exposed and easily reacts with electrophilic compounds [[60], [61], [62]]. It is in this context noteworthy that 2,3-dimercaptopropanesulfonic acid inhibited all selenoproteins but not GR, suggesting that this small molecule may access the Sec residue in GPX4 as well, and may thus possibly be a pan-selenoprotein inhibitor, although we have not yet tested it against other selenoproteins. Still, the evaluation of the inhibitory activity of the different compounds on other selenoproteins remains an important step in efforts to discover specific GPX inhibitors.

Following triage assays, auranofin (classically known as a TXNRD1 inhibitor) and five novel compounds were identified as GPX1/GPX2-specific inhibitors. Of these six compounds, auranofin, omapatrilat, and tenatoprazole showed significant shifts in the melting temperature of GPX1 and can thus be considered direct binders of the enzyme. The two cephalosporins, cefoxitin sodium salt, and ceftibuten did not show any thermal stabilization. The final three compounds do not share structural similarity, nor overlap in mechanism of action. Omapatrilat was developed as a dual neutral endopeptidase (NEP) and angiotensin-converting enzyme (ACE) inhibitor that has been experimentally assessed as a hypertension treatment, while tenatoprazole was a clinical candidate for peptic ulcer and reflux functioning as a proton pump inhibitor. Based on our findings, targeting of GPX1 and/or GPX2 could also be considered as a potential off-target mechanism of action for these compounds.

The GPX2 isoform of human selenoprotein GPXs is found in epithelial cells and has similar substrate specificity with GPX1, but not as much with GPX4 [8]. Additionally, GPX1 and GPX2 both form homotetramers and share similarities in structural homology, although GPX2 has a much slower turnover rate. All GPX1 inhibitors found here also showed similar potency against GPX2, but it should be noted that because the activity of GPX2 is much lower than that of GPX1, our assays required a 25-fold higher concentration of GPX2 to measure activity. GPX2 was still effectively inhibited at lower enzyme:compound ratios, showing that GPX2 was efficiently inhibited. It remains to be seen whether cross-inhibition of GPX1 and GPX2 will prove to be a hurdle for drug development efforts seeking specific inhibitors of GPX1 or GPX2. Furthermore, any cellular effects of the compounds identified here as GPX1 inhibitors must also be considered as resulting from GPX2 inhibition, or inhibition of both enzymes, depending on their respective expression profiles in a given cell or tissue.

We identified 13 novel biochemical inhibitors of GPX4. These inhibitors proved less promiscuous in our suite of assays, showing little to no cross-inhibition with GPX1, GPX2, or TXNRD1. Although the final high-quality inhibitors were varied in mechanism of action and structure, several categories of structurally similar hits were identified, helping to validate the assay and pipeline. Unsurprisingly considering the affinity of GPX4 for phospholipid hydroperoxides, the largest category of inhibiting compounds contained fatty acids and surfactants. These tended to inhibit primarily at top doses and were not considered high-quality hits, however several of the final hits, namely lusutrombopag and pranlukast, contain longer carbon chains. Of the more drug-like classes, these included three NS3-4A serine protease inhibitors produced for treatment of hepatitis C: simeprevir, grazoprevir, and paritaprevir. Furthermore, the Bcl-2 family protein inhibitors, navitoclax and venetoclax, both showed potent GPX4 inhibition. The final set of GPX4 inhibitors included GSK-3965 and CDIBA, liver X receptor (LXR) agonist and a cytosolic phospholipase A2 (cPLA2) inhibitor, respectively. The remaining top high-quality hits, namely brilanestrant and VU0661013 were considered singleton hits and remain structurally different from the other compounds. Important to consider when assessing these hits is our choice to use the substrate CHP in our assay. This helped to facilitate the difficulties of HTS, so these hits have not been assessed using other lipid hydroperoxides as GPX4 substrates (and such a HTS-amenable biochemical assay has not yet been reported). If GPX4 inhibition contributes to the pharmacological effects of any of these compounds when used *in vivo* remains to be investigated.

The purpose of this study was to establish an HTS discovery pipeline for GPXs and to evaluate the workflow for identifying specific probes inhibiting GPX1 and GPX4. Though the compounds screened have other established targets and are known to be promiscuous, and were selected for that purpose, the identification of auranofin, a known GPX1 inhibitor, using these assays helps to validate the screening process. During this pilot, many compounds were identified that interfere with GR and TXNRD1 in secondary assays, demonstrating the relevance of these follow-ups to exclude false-positive hits and more general Sec-targeting activity. In total, 20 novel GPX inhibitors, two of which were pan-GPX inhibitors, five inhibited GPX1 (and GPX2) and 13 specifically inhibited GPX4. Furthermore, the overlap in compounds that inhibit GPX1 and GPX2 suggests the importance of assaying GPX2 inhibition, and possibly other GPX isoforms, to fully characterize GPX1 inhibition. With our studies confirming several compounds as inhibitors of GPXs, the effects and possible mechanisms of action *in vivo* for these compounds should be assessed next, with a focus on GPX inhibition. This is especially important considering that the chemical libraries utilized here encompassed compounds having previously confirmed pharmacological activities and presumed mechanisms of action. GPX inhibition has, however, typically not been considered as a possible activity for most of these compounds.

Our study provides a basis for future identification, development, and characterization of novel GPX inhibitors for the potential treatment of malignancies by establishing a series of robust assays designed for detecting and triaging hits from high-throughput small molecule library screens.

## Author contributions

DMC, ESJA, and MDH conceived the study and designed the experiments. DMC performed the experiments. DMC and QC produced recombinant proteins, including GPXs and TXNRDs. JT and CKT assisted with automation and library screens. HG and MS analyzed the data. DMC wrote the final manuscript, which was revised by QC, HG, JT, CKT, MS, ESJA, MDH.

## Funding

This work was partially supported by the intramural research programs of the 10.13039/100006108National Center for Advancing Translational Sciences, National Institutes of Health. DMC was supported through a joint NIH-Karolinska Insitutet graduate training program. ESJA acknowledges funding from 10.13039/501100004047Karolinska Institutet, The Knut and Alice Wallenberg Foundations (KAW 2019.0059), The 10.13039/501100002794Swedish Cancer Society (21 1463 Pj), The 10.13039/501100004359Swedish Research Council (2021–02214), National Laboratories Excellence program under the National Tumor Biology Laboratory project (2022–2.1.1-NL-2022-00010) and the Hungarian Thematic Excellence Programme (TKP2021-EGA-44) and The 10.13039/501100011019National Research, Development, and Innovation Office (NKFIH) grant ED_18-1-2019-0025.

## Declaration of competing interest

ESJA and QC are shareholders in Selenozyme AB, a company selling recombinant selenoproteins.

## Data Availability

The datasets presented in this study can be found in online repositories. The repository/repositories using accession number(s) 1845191 for the GPX1 HTS and 1845192 for the GPX4 HTS can be found in the PubChem BioAssay on: https://www.ncbi.nlm.nih.gov/.

## Abbreviations

AUC: area under the curve
BSA: bovine serum albumin
BSO: buthionine sulfoximine
CHP: cumene hydroperoxide
CRC: concentration-response curves
FIN: ferroptosis inducer
GPX: glutathione peroxidase
GR: glutathione reductase
GSH: reduced glutathione
GSSG: oxidized glutathione
HTS: high-throughput screening
mBBr: monobromobimane
MSA: mercaptosuccinic acid
NADPH: reduced nicotinamide adenine dinucleotide phosphate
NSCLC: non-small-cell lung cancer
PDAC: pancreatic ductal adenocarcinoma
ROS: reactive oxygen species
RSL3: (1S,3R)-RSL3
Sec: selenocysteine
TES Buffer: Tris-HCl, EDTA and NaCl
TE buffer: Tris-HCl, EDTA
TXNRD1: thioredoxin reductase 1
qHTS: quantitative high-throughput screening
