# Supplementary material for: Development of an assay pipeline for the discovery of novel small molecule inhibitors of human glutathione peroxidases GPX1 and GPX4
Source: Redox Biol. 2023 May 16;63:102719. doi: 10.1016/j.redox.2023.102719 (PMC10220285; doi:10.1016/j.redox.2023.102719)
Supplement: Multimedia component 1 [file mmc1.docx]

**Supplementary figures and tables:**

**
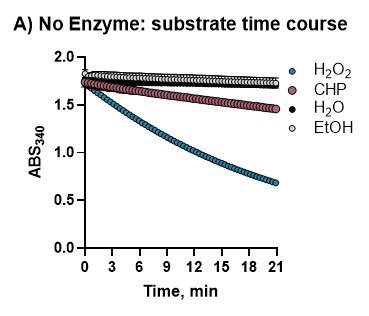
**

**Supplementary figure 1: Background activity of substrates in GR-coupled GPX activity.** A) NADPH consumption of hydrogen peroxide (H2O2), cumene hydroperoxide in EtOH, water, and EtOH in a no enzyme control of GPX activity assays. Data are changes to ABS340 over time and presented as mean ± s.d. of n = 4 replicates.

**Supplementary figure 2: Validation of GR qHTS primary assay.** A) GR dilution and time course of GR activity assay, red denotes optimized concentration of 1 nM; B) Time course at various GSSG concentration in GR activity assay, red denotes optimized concentrations of 1 mM GSH; C) 1536-well plate performance of optimized GR activity assay, average Z’ for whole plate was 0.77.

**Supplementary figure 3: Validation of monobromobimane GPX secondary assay.** A) GSH linearity with 500 μM MBBr; B) Time course for GPX1, GPX4, and no enzyme control; C) 384-well plate performance of optimized MBrB GPX1 activity assay, average Z’ for whole plate was 0.49; E) 384-well plate performance of optimized MBrB GPX4 activity assay, average Z’ for whole plate was 0.47.
